# Visualization of ^14^CO_2_ gas fixation by plants

**DOI:** 10.1007/s10967-018-6119-3

**Published:** 2018-08-24

**Authors:** Ryohei Sugita, Natsuko I. Kobayashi, Keitaro Tanoi, Tomoko M. Nakanishi

**Affiliations:** 1 Graduate School of Agricultural and Life Sciences, The University of Tokyo, 1-1-1, Yayoi, Bunkyo-ku, Tokyo, 113-8657 Japan; 2 PRESTO, Japan Science and Technology Agency (JST), 4-1-8 Honcho, Kawaguchi, Saitama 332-0012 Japan

**Keywords:** Real-time radioisotope imaging system, ^14^CO_2_ gas, Soybean, Carbon fixation, Orientation of photosynthate movement

## Abstract

Using the real-time radioisotope imaging system (RRIS), we present the carbon dioxide gas fixation process of a soybean plant applying the ^14^C-labeled gas. When ^14^CO_2_ gas was supplied to the selected mature leaf, the fixed carbon, photosynthate, was transferred and accumulated to the younger leaves preferentially within 24 h. When ^14^CO_2_ gas was supplied to the younger leaves, fixed carbon was hardly moved. In the case of the pods, fixed ^14^CO_2_ gas in the leaf was preferentially transferred to the closest pod.

## Introduction

Carbon constituting the plant tissue is derived from the fixation of the carbon dioxide in air by photosynthesis. Since photosynthesis is one of the main activities of the plant, many researches were conducted to analyze the mechanism of photosynthesis. Discovery of the Calvin-Benson cycle is the essential and representative one, how sucrose is produced after carbon fixation. Although there are many studies to analyze the mechanism of photosynthesis, the actual movement of photosynthate in an intact plant was not reported well.

To know the movement of the photosynthate in a plant, employment of non-destructive imaging method, which selectively visualizes photosynthate, is necessary. However, the real-time imaging system using radioisotopes, especially applying radioisotope labeled gas, for plant sample has not been well developed. We have been developing the real-time imaging system, both for macroscopic and microscopic imaging [1–9].

Principally, the radioisotope imaging system is comprised of a scintillator and a CCD camera. The radiation from the plant, after application of a radioisotope, is converted to light by the scintillator and the radioisotope image of the plant is taken by a highly sensitive CCD camera. Since plants live on inorganic ions, many kinds of element movement were visualized using this system [5–9].

To image carbon dioxide fixation, the next problem was what kind of radioisotope was preferable for this kind of imaging. There are two radioisotope candidates for carbon imaging, ^14^C (half-life: 5730 y) and ^11^C (half-life: 11 min). ^11^C is a positron emitter and is well used for diagnosis in medical field known as PET (Positron Emission Tomography). Since the half-life of ^11^C is too short to apply for this kind of research, ^14^C was employed to label carbon dioxide gas. The ^14^CO_2_ gas was prepared by mixing ^14^C-labeled sodium hydrogen carbonate with 2-hydroxypropanoic acid (lactic acid). Then, the emitted ^14^CO_2_ gas was introduced to a plastic bag covering the portion of the plant tissue.

A soybean plant was selected for this research, since this is an important crop and also because of the size. An Arabidopsis was employed for visualizing the element movement [3–6]. It was too small to study the accumulation of the radioisotopes in detail. Since we are targeting the pod for further development of this study besides leaves, stems or roots, the relatively large plant was preferable.

This is the first report to visualize the movement of photosynthate by applying ^14^CO_2_ gas to the soybean plant.

## Materials and methods

### Plant sample preparation

After washing with water well, the seeds of *Glycine max*. cv. Enrei were germinated in vermiculite. After 3 days, the seeds were transferred to water culture solution, 1/2 Hoagland culture solution, and grown under 16 h light/8 h dark condition at 25 °C, with about 60% of humidity. After 20–55 days, the plants were transferred to the imaging box and the whole part of the plant was fixed on a scintillator (10 cm × 20 cm), covered with a thin polyethylene film (10 µm, in thickness) by a tape so that the contact of the plant to the scintillator was maintained during the imaging. The vinyl bag was prepared to cover the whole plant or the targeted tissue of the plant. The bag was set to the tissue tightly by a tape and clay so that the air within the bag did not leak.

### Application of ^14^CO_2_

A 100 µl of NaH^14^CO_3_ (2 µmol, 4 MBq) solution was added to a 200 µl of lactic acid solution (10 mmol) in a 5 ml vessel. From this vessel, a tube was coming out and connected to the vinyl bag covering the plant to introduce the ^14^CO_2_ gas. When all the chemical reaction was confirmed to be completed, a 30 ml syringe was driven into the vessel and pushing the syringe, the air was sent to the vessel 4 times to transfer the ^14^CO_2_ gas completely to the vinyl bag. After 30 min of the ^14^CO_2_ gas supply, the vinyl bag was removed from the plant and was used for the successive imaging treatments.

### Imaging ^14^C in the plant

In the case of real-time imaging, the plants treated with ^14^CO_2_ gas growing in a pot was transferred and set in a big imaging box (120 cm × 140 cm) equipped with a CCD camera. Then, the radiation from the sample was converted to the light by a CsI(Tl) scintillator deposited to 100 µm, in thickness, on a fiber optic plate (FOS). The imaging box was kept in dark for 15 min while taking the image by a CCD camera (C8600-04, Hamamatsu Photonics, Co.). After taking the image, the imaging box was kept light for 15 min so that the physical activity, i.e. photosynthesis of the plant, was kept active. This 15 min dark/15 min light cycle was repeated 16 times, after ceasing the supply of the ^14^CO_2_ gas. To analyze the radioactivity intensity, regions of interest (ROIs) were set at test plant in each sequential image. Furthermore, ROIs were set in the place where there was not a test plant for calculating background. And then, net value was calculated by subtracting background from test plant value. The ROIs were calculated using ImageJ software.

In the case of the imaging using an imaging plate (IP: 20 cm × 40 cm, BAS IP-MS, GE Healthcare UK), the plant with roots was harvested from the pot, after 8 or 24 h of the ^14^CO_2_ gas supply. Then the plant was covered with a thin polyethylene film (10 µm, in thickness) and was exposed to the IP in a cassette for 30 min. The ^14^C image in the plant was obtained from scanning the IP by a scanner (FLA5000 image analyzer, FUJIFILM Co.) and was taken by a computer equipped with Image Gauge version 4.0 (FUJIFILM Co.)

## Results

**Fig. 1:**
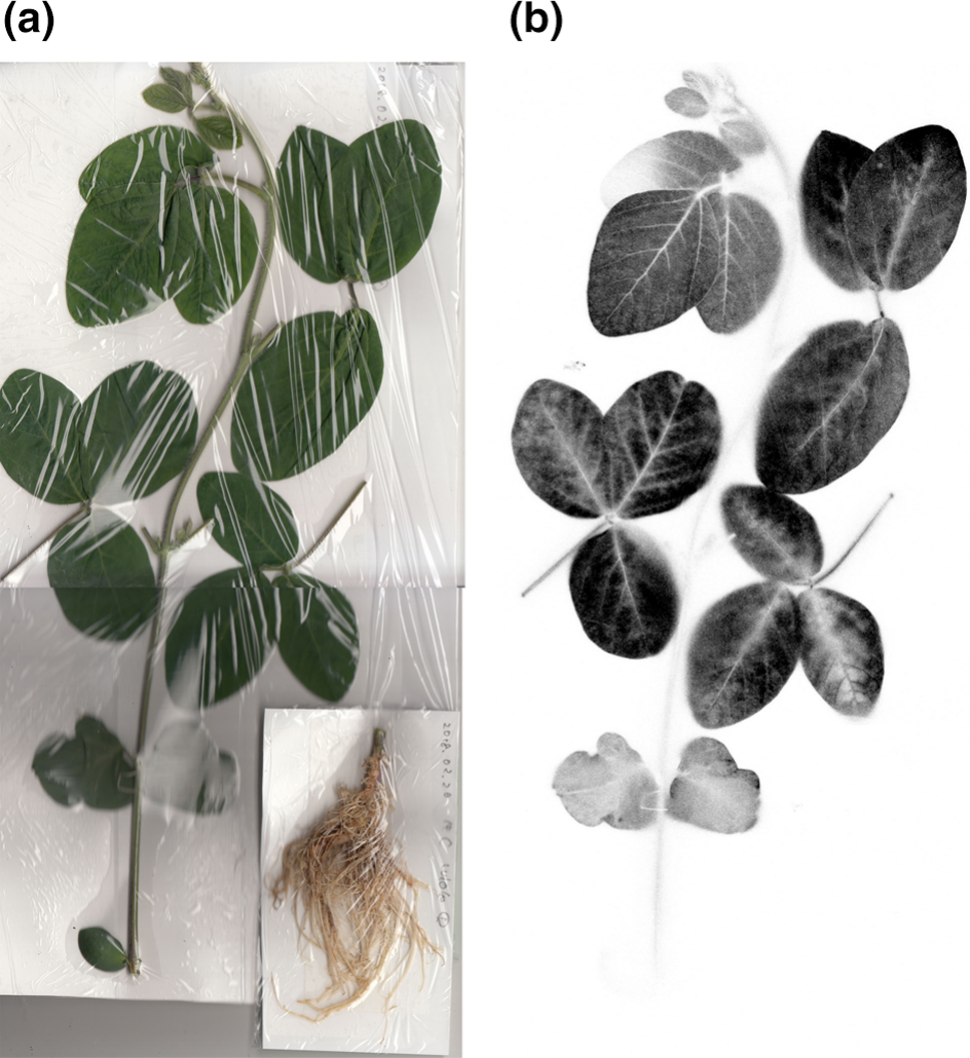
Photosynthate image in a plant. The ^14^CO_2_ gas was produced and exposed to a soybean plant for 30 min. Then, the plant was exposed to an imaging plate (IP) for 30 min. **a** A picture of the plant. **b** The radiograph of the image in the IP. (Color figure online)

When ^14^CO_2_ gas was supplied for 30 min to the whole plant grown for 40 days after germination, the IP image was taken to get the distribution of the ^14^C-labeled compounds within the plant. It was found that ^14^C was distributed uniform among the developed leaves, suggesting that similar amount of photosynthate was produced by 30 min photosynthesis at each leaf. Figure 1 is the image of the plant obtained from IP. In the case of the root, there was not any ^14^C observed in the image. It seemed that most of the photosynthate produced in leaves was remaining at the site where the photosynthesis occurred, therefore, it was not yet transferred to the roots.

**Fig. 2:**
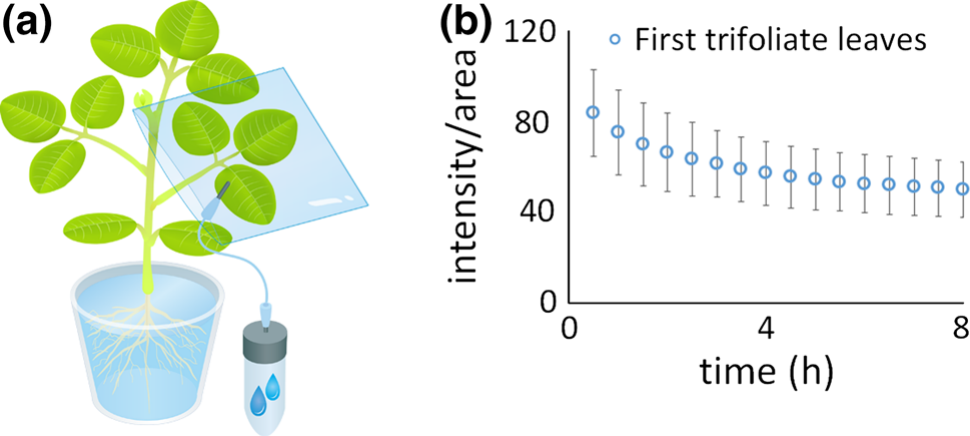
Photosynthate transferring manner. The ^14^CO_2_ gas was produced and exposed to the trifoliate leaves for 30 min and then the real-time imaging was performed. The radioactivity intensity of the trifoliate leaves was monitored from the intensity of the image taken by the real-time imaging system after ^14^CO_2_ gas was supplied. **a** Schematic illustration of ^14^CO_2_ gas supply to the trifoliate leaves. **b** The relative radioactivity intensity curve of the trifoliate leaves with time based on the sequential images, after the ^14^CO_2_ gas was supplied. Mean ± standard deviation of four plants are presented. (Color figure online)

To know the transfer movement of photosynthate from one tissue to the other tissue, real-time imaging was performed after supplying the ^14^CO_2_ gas to the specific tissue. When the specific trifoliate leaves were chosen for the gas supply and the radioactivity changes of this trifoliate leaves as well as the neighboring tissue were obtained by the imaging system, the transferring amount of photosynthate with time was able to monitor from the intensity of the image, since the intensity of the image corresponds to the radioactivity amount. As is shown in Fig. 2, all of the photosynthate amount produced in the original trifoliate leaves was gradually decreased with time and became plateau after about 4 h.

**Fig. 3:**
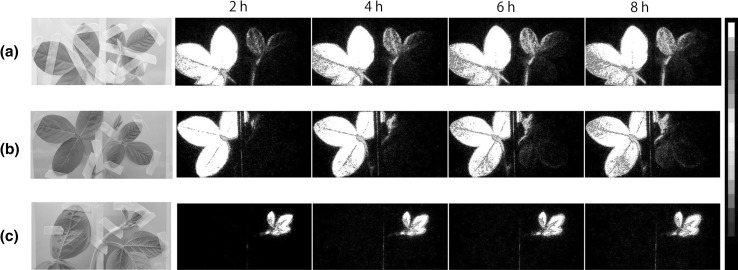
The photosynthate movement image taken by the real-time imaging system. The ^14^CO_2_ gas was supplied to the selected tissue for 30 min and the movement of the photosynthate was monitored by the real-time imaging system. The lap-time images after the ^14^CO_2_ gas supply were shown in the figures. **a** ^14^CO_2_ gas was supplied to the trifoliate leaves to the plant with younger leaves. **b** ^14^CO_2_ gas was supplied to the trifoliate leaves to the plant with younger leaves and meristem tissue. **c** ^14^CO_2_ gas was supplied to the younger tissue of the plant. The detection time for each image was set to 15 min. In each image, signal intensity is indicated by pseudo color (white represents high intensity). (Color figure online)

The photosynthate movement image taken by our imaging system was shown in Fig. 3. The ^14^CO_2_ gas was supplied to the selected tissue of a 20-day old plant for 30 min and the movement of the ^14^C images was taken. As is shown in the figure, there was a specific route for the photosynthate produced in the trifoliate leaves. The metabolites were transferred preferentially to the youngest leaves (Fig. 3a). Even to the very small developing tissue of the young leaves, the photosynthate was selectively transferred to this meristem tissue and was accumulated (Fig. 3b). However, when the ^14^CO_2_ gas was supplied only to the youngest leaves including meristem part, the photosynthate produced at this tissue remained at this site and hardly moved to the other tissue (Fig. 3c).

**Fig. 4:**
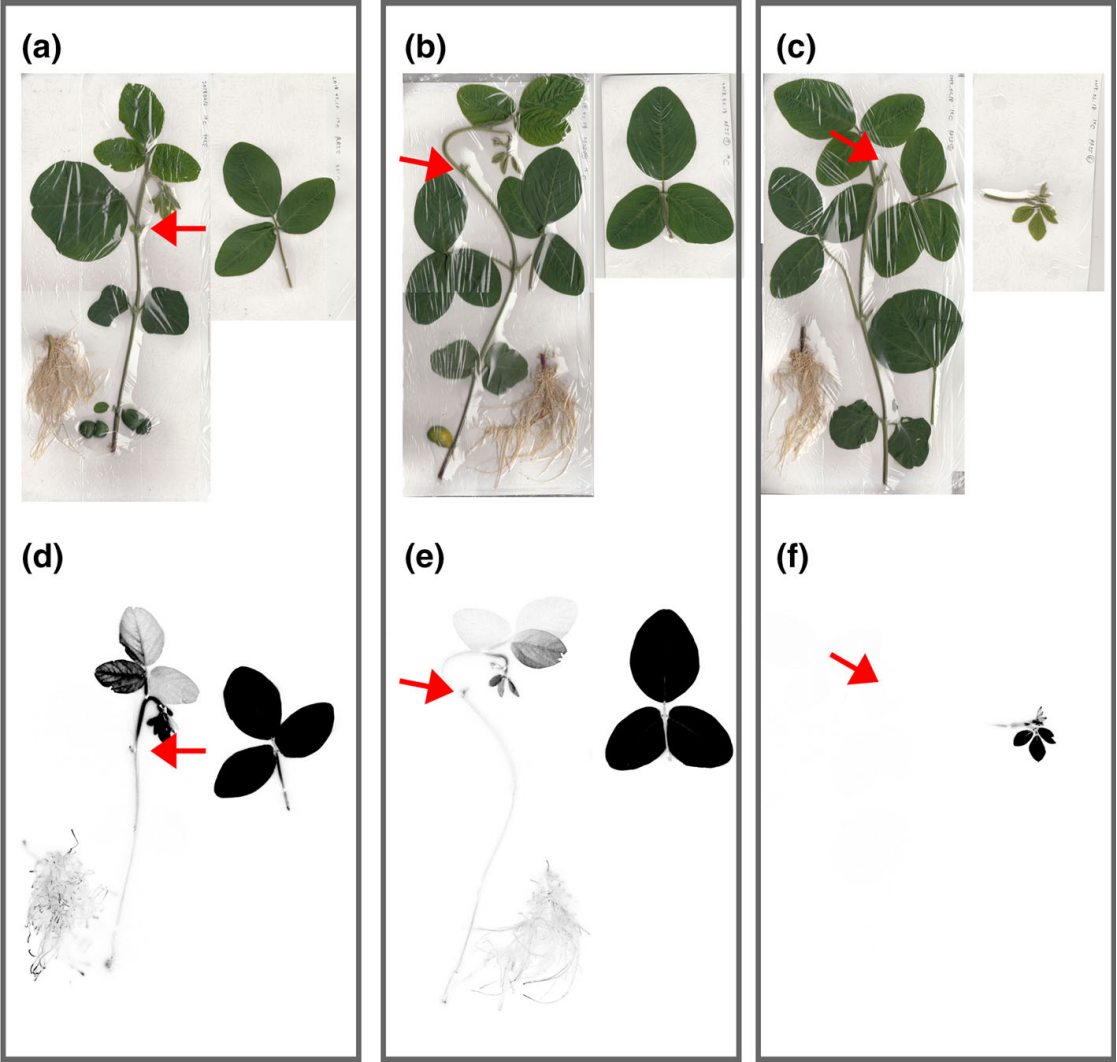
Photosynthate profile in a plant. After 24 h of the ^14^CO_2_ gas supply to the trifoliate leaves, this trifoliate leaves were removed from the plant and was exposed to an IP. The upper figures (**a**–**c**) are the pictures corresponding to the lower IP images and the lower figures (**d**–**f**) are the images obtained from the IP. The ^14^CO_2_ gas was supplied to the expanded trifoliate leaves (**a**, **b**, **d** and **e**) and youngest tissue (**c** and **f**). To take lower back ground of the IP images, the leaves or tissue were disconnected from the plant before taking IP images. The sites of the plant where these tissues were cut off were shown by the arrows (**d**–**f**). (Color figure online)

After 24 h of the ^14^CO_2_ gas supply, the plants were harvested and were exposed to the IP plates for 30 min. Figure 4 showed the image of these plants taken by the IP. The upper figures (Fig. 4a–c) are the pictures corresponding to the lower IP images and the lower figures (Fig. 4d–f) are the images obtained from the IP. In the case of Fig. 4a, b, d and e, the ^14^CO_2_ gas was supplied to the trifoliate leaves. Figure 4c and f show the image when the ^14^CO_2_ gas was supplied to the youngest tissue. Since the leaves supplied with ^14^CO_2_ gas emits higher radiation compared to the other tissue due to the remaining ^14^C-labeled compounds, the background level of the image was increased when all the plant was exposed to the same IP. Therefore, the leaves were disconnected from the plant and the images of the cut off leaves, and the rest of the plant were taken by the different IP. The original growing site of these trifoliate leaves was indicated by an arrow in the picture.

The IP images (Fig. 4d, e) showed that when the ^14^CO_2_ gas was supplied to the expanded trifoliate leaves, most of the photosynthate was preferentially moved to the youngest tissue and only small amount of the photosynthate was moved to the roots or to the other tissue, indifferent to the position of the trifoliate leaves developed. However, when the ^14^CO_2_ gas was supplied to the youngest tissue (Fig. 4f), all of the photosynthate produced at this site was remained at this site and the movement to the other tissues was not shown, including roots.

**Fig. 5:**
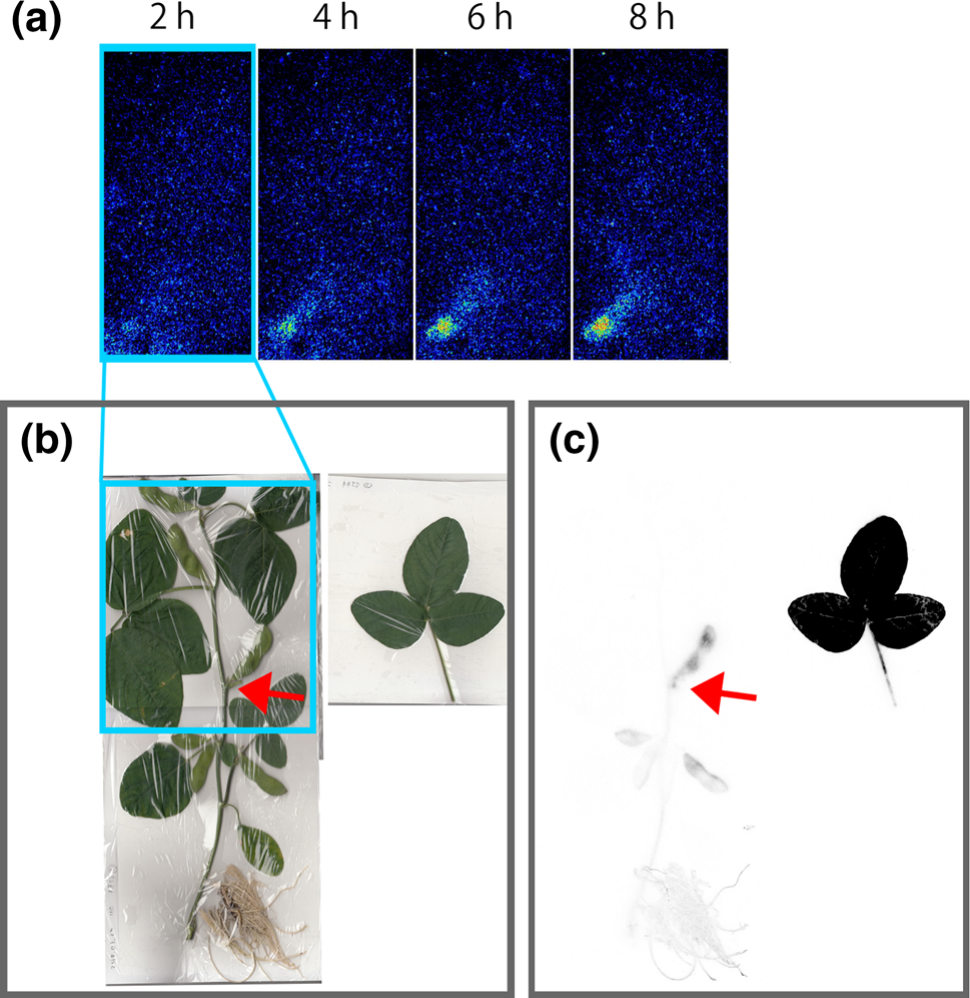
Photosynthate movement from leaves to a pod. The ^14^CO_2_ gas was supplied to the expanded trifoliate leaves and **a** the photosynthate accumulation images with time was shown. The sequential images were taken by real-time imaging system. The photosynthate accumulation image in the plant was taken by an IP. The picture of the plant after real-time imaging (**b**) and corresponding IP image (**c**). The treated trifoliate leaves were disconnected from the plant and the disconnected site was shown by an arrow (**b** and **c**). (Color figure online)

To know the orientation of the photosynthate movement from the leaves in the plant developing the pods, the plants after 55 days of the germination was selected. The ^14^CO_2_ gas was supplied for 30 min to the trifoliate leaves grown at the site close to the pod. The accumulation images obtained from the real-time imaging system and the IP image taken after 8 h was shown in Fig. 5. As is shown in Fig. 5a, the accumulation of the photosynthate from the trifoliate leaves was observed at the closest pod and the accumulation amount was still increasing even after 8 h of the ^14^CO_2_ gas supply. Using the IP, the preferential accumulation to the pod was confirmed and the accumulation of the photosynthate was not observed at the other tissues (Fig. 5b, c). In this case, the trifoliate leaves supplied with the ^14^CO_2_ gas was disconnected from the plant to decrease the background level of the image and was exposed to the other IP, similar treatment performed was also shown in Fig. 4. The disconnected site of the trifoliate leaves was shown by arrows (Fig. 5b, c). Though there were several pots developed in the plant, the accumulation of the photosynthate was not shown at those developed at higher position of the treated trifoliate leaves but at the closest pod. However, small amount of the photosynthate accumulation was shown at the pods grown under the applied leaves as well as in roots (Fig. 5c). There was not any accumulation of the photosynthate to the pods developed at the higher position indicated that there was not any supply of the metabolites to the younger tissue as shown in Fig. 4. It was suggested that photosynthate was not transferred long distance when pods were developed, since the photosynthate supply to the pod was mainly from the neighboring leaves.

## Discussion

One of the most important activities of the plant is photosynthesis. However, little is known about the carbon fixation and the movement especially the route of the photosynthate transfer among the plant tissue. Using isotopically labeled carbon dioxide is the only method to visualize the photosynthate. There are two elements for labeling the carbon dioxide gas, either C or O. The available radioisotope for O is ^15^O (half-life: 2 min, a positron emitter), and for C is ^14^C (half-life: 5730 y, beta-ray emitter), or ^11^C (half-life: 11 min, a positron emitter). In the case of imaging the carbon fixation process, the half-life should be longer than several hours. Therefore, ^14^C was selected to label the carbon dioxide gas. However, the energy of ^14^C is very low, with maximum energy of 156 keV, the absorption of the beta-ray was not able to neglect, including self-absorption. Based on the decreasing manner of the radiation from the image reported before [10], the real-time imaging using our system was performed. In the case of the trifoliate leaves (0.2 mm in thickness) and in the case of the pod (10 mm in thickness), radiation from the tissue was estimated to decrease to about 0.6% and no more than 0.3%, respectively. Therefore, the real-time image of the carbon fixation was to show the qualitative analysis of the transferring route of photosynthate.

Figure 1 showed that about the same amount of the photosynthate was produced using the image by an IP. Based on the similar amount of the photosynthate accumulation, the transferring manner of the photosynthate from one trifoliate leaves was studied using the real-time imaging system we have been developing. The Fig. 2b was obtained from the intensity of the images in the treated trifoliate leaves. The decreasing curve of the relative intensity in the treated leaves showed that within 8 h most of the transferring movement of the photosynthate seemed to be ceased. Therefore, the other real-time imaging was performed until 8 h.

The route of the photosynthate from trifoliate leaves was clearly shown by the images taken both by the real-time imaging system and by an IP. Using 20-day old plants, the photosynthate was preferentially moved to the youngest tissue and was accumulated, in different to the position of the trifoliate leaves developed. There are many kinds of the tissue with different developmental stage in one plant, therefore, it was suggested that it is very important to protect and develop the youngest tissue, by transferring the photosynthate.

However, how carbon is fixed and forming the new tissue is not known well in detail. For example, we found that the route to transfer the photosynthates is different among the tissues, suggesting that they are not only one way for translocation of the chemicals according to the simple rule, from source to sink.

Here, we could present the photosynthate movement within a soybean plant by applying ^14^CO_2_ gas to the plant tissue, using both by the real-time imaging system we have developed and by using an IP. Since the application of the radioisotope imaging method is the only way to visualize the movement of chemicals within an intact plant, this kind of imaging method is expected to develop further.

## Conclusions

1. When ^14^CO_2_ gas was supplied to the whole up-ground part of the plant, the accumulation amount of the photosynthate was about the same among the trifoliate leaves.
2. The fixed carbon, photosynthate, of the expanded trifoliate leaves was transferred and accumulated to the younger leaves preferentially within 24 h.
3. When ^14^CO_2_ gas was supplied to the younger leaves, fixed carbon was maintained at the same tissue and hardly moved.
4. In the case of the pods, fixed ^14^CO_2_ gas in the leaf was preferentially transferred to the closest pod.
5. Using the real-time radioisotope imaging system (RRIS), ^14^CO_2_ gas fixation process as well as the orientation of the photosynthate transfer in a soybean plant was visualized, applying the ^14^C-labeled gas produced by the chemical reaction.
